# Cardiovascular Risk Prediction Models and Scores in the Era of Personalized Medicine

**DOI:** 10.3390/jpm12071180

**Published:** 2022-07-20

**Authors:** Areti Sofogianni, Nikolaos Stalikas, Christina Antza, Konstantinos Tziomalos

**Affiliations:** 1 First Propedeutic Department of Internal Medicine, Medical School, Aristotle University of Thessaloniki, AHEPA Hospital, 54636 Thessaloniki, Greece; aretisofo@hotmail.com; 2 Department of Cardiology, Medical School, Aristotle University of Thessaloniki, AHEPA Hospital, 54636 Thessaloniki, Greece; nstalik@gmail.com; 3 Third Department of Internal Medicine, Medical School, Aristotle University of Thessaloniki, Papageorgiou Hospital, 54636 Thessaloniki, Greece; kris-antza@hotmail.com

**Keywords:** cardiovascular risk, prediction, equation, SCORE, pooled cohort equations, personalized medicine

## Abstract

Cardiovascular disease (CVD) is the leading cause of death worldwide. Management of cardiovascular risk factors, particularly hypertension and dyslipidemia, has been shown to reduce cardiovascular morbidity and mortality. However, current guidelines recommend adjusting the intensity of blood pressure- and lipid-lowering treatment according to the cardiovascular risk of the patient. Therefore, cardiovascular risk prediction is a sine qua non for optimizing cardiovascular prevention strategies, particularly in patients without established CVD or type 2 diabetes mellitus (T2DM). As a result, several cardiovascular risk prediction equations have been developed. Nevertheless, it is still unclear which is the optimal prediction risk equation. In the present review, we summarize the current knowledge regarding the accuracy of the most widely used cardiovascular risk prediction equations. Notably, most of these risk scores have not been validated in external cohorts or were shown to over- or underestimate risk in populations other than those in which they derive. Accordingly, country-specific risk scores, where available, should be preferred for cardiovascular risk stratification.

## 1. Introduction

Cardiovascular disease (CVD) is the leading cause of death worldwide [1]. Management of cardiovascular risk factors, particularly hypertension and dyslipidemia has been shown to reduce cardiovascular morbidity and mortality [2,3]. However, current guidelines recommend adjusting the intensity of blood pressure- and lipid-lowering treatment according to the cardiovascular risk of the patient [4,5]. Therefore, cardiovascular risk prediction is a sine qua non for optimizing cardiovascular prevention strategies, particularly in patients without established CVD or type 2 diabetes mellitus (T2DM) [4,5]. As a result, several cardiovascular risk prediction equations have been developed. Nevertheless, it is still unclear which is the optimal prediction risk equation.

In the present review, we summarize the current knowledge regarding the accuracy of the most widely used cardiovascular risk prediction equations (Table 1).

## 2. Systematic Coronary Risk Evaluation (SCORE)

**Figure 1:**
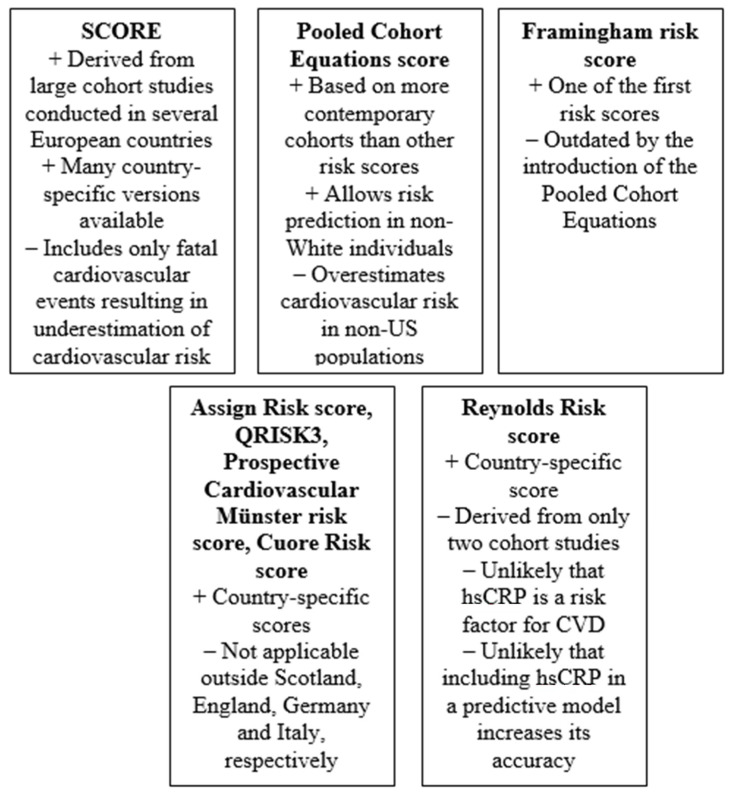
Key advantages and pitfalls of most relevant risk scores (hsCRP: high-sensitivity C-reactive protein; CVD: cardiovascular disease).

SCORE (Systematic Coronary Risk Evaluation) (Figure 1) predicts the 10-year risk of cardiovascular mortality and was developed from 12 European cohort studies (n = 205,178) with 7934 cardiovascular deaths [6,7]. SCORE takes into account the following parameters: age, sex, systolic blood pressure (SBP), total cholesterol (TC) and smoking [6,7]. The age range is 40 to 65 years old, and patients with established CVD or T2DM are excluded [6,7]. There are three versions of SCORE for low-, high- and very-high-risk countries, respectively, as well as country-specific versions [6,7].

A number of studies compared the predictive ability of SCORE with other risk equations. In the Hoorn Study (n = 1482), SCORE was more accurate than the Framingham and UK Prospective Diabetes Study risk equations in patients with normal glucose tolerance [8]. Notably, SCORE was equally precise for estimating risk in patients with normal glucose tolerance and impaired glucose tolerance, but less accurate in patients with T2DM [8]. In a smaller study from Spain (n = 608), both SCORE and Framingham overestimated cardiovascular risk, but the former was more accurate [9]. In another study (n = 1344), SCORE had better specificity than the risk chart developed by the European Society of Hypertension, but the latter was more sensitive [10]. In the Third National Health and Nutrition Examination Survey (n = 5999), the SCORE and the Framingham risk prediction equation discriminated cardiovascular mortality risk equally well [11]. In a large Dutch cohort study (n = 39,719), the SCORE and the Framingham risk prediction equation had similarly good discrimination, but were both inadequately calibrated [12]. In contrast, in the Atherosclerosis Risk In Communities (ARIC) Study (n = 14,343), the SCORE showed worse discrimination than the Framingham equation, possibly because this study was performed in the US [13].

A limited number of studies evaluated the concordance between the SCORE and markers of subclinical atherosclerosis or target-organ damage. In a cohort of 190 patients without symptoms of coronary heart disease (CHD), there was a strong correlation between SCORE and the results of coronary computed tomographic angiography [14]. In another study, the presence of carotid atherosclerotic plaques, urine albumin/creatinine ratio, left ventricular mass and pulse wave velocity predicted cardiovascular risk independently from SCORE and combining the two methods improved the accuracy of SCORE [15].

An important limitation of the SCORE is that it is not applicable in patients older than 65 years. Accordingly, a version of SCORE for older patients has been developed and was evaluated in the European Prospective Investigation of Cancer Norfolk study (n = 6590) [16]. Although this version of SCORE was accurate in predicting cardiovascular mortality, its discriminative action was inadequate [16]. More specifically, it overestimated cardiovascular mortality risk in subjects aged 65–69 years and in normotensive subjects, whereas it underestimated cardiovascular mortality risk in hypertensive patients and in subjects 70–79 years-old [16].

The major advantages of SCORE are that it is derived from large cohort studies conducted in several European countries and that there are many country-specific versions derived from local data, which are expected to be more accurate. The major disadvantages is that it includes only fatal cardiovascular events and might therefore underestimate the total cardiovascular risk.

## 3. Pooled Cohort Equations Calculator

The Pooled Cohort Equations Calculator, first published in the 2013 American College of Cardiology and American Heart Association guidelines, uses simple parameters such as sex, age, TC, high-density lipoprotein cholesterol (HDL-C), SBP, treatment for hypertension, history of T2DM and smoking status to predict the 10-year risk of a first hard cardiovascular event, defined as nonfatal myocardial infarction (MI), CHD death and fatal or nonfatal stroke [17]. The rationale for the development of this equation was to replace the widely used Framingham risk score because the latter was derived from only White populations and only evaluated the risk of CHD [17]. In contrast, the Pooled Cohort Equations Calculator was created using data from a wider range of studies in both White and African American populations [18,19,20,21,22].

The first validation of the Pooled Cohort Equations score was performed in 2014 from a cohort study, using data from a population similar with the one from which the score was derived [23]. The results showed that the observed and predicted 5-year cardiovascular risk for participants with 10-year predicted risk < 7.5% was similar, while for those at risk ≥ 7.5%, the Pooled Cohort Equations overestimated risk [23]. Ensuing studies including analyses from the Women’s Health Study, the Physicians’ Health Study, the Women’s Health Initiative Observational Study and the National Cardiovascular Data Registry Practice Innovation and Clinical Excellence registry were generally in accordance with this first publication, confirming that the Pooled Cohort Equations score overestimates cardiovascular risk, particularly in elderly subjects [24,25,26,27,28,29,30]. Results from a multi-ethnic cohort show that the observed overestimation is also highest among Chinese (especially for men) and lowest in White women and Hispanic men [31]. When the Pooled Cohort Equations score was evaluated in non-Hispanic White and Black people as well as in Mexican Americans, the prediction of 10-year atherosclerotic CVD mortality was accurate in non-Hispanic White and Black men, but not in women [32].

The Pooled Cohort Equations score was also evaluated for other outcomes, except cardiovascular mortality. It has been reported that this score could be a useful tool to predict and stratify 1-year risk of recurrent stroke and total cardiovascular events in patients with acute ischemic stroke or transient ischemic attack [33]. Compared with the Framingham risk score, the Pooled Cohort Equations score provided a better estimate of racial differences in vascular function and structure [34]. Moreover, a higher score using this equation was found to be associated with increased 24h variability of blood pressure [35] and also with worse health-related quality of life [36].

The major advantages of the Pooled Cohort Equations score is that it is based on more contemporary cohorts than other risk prediction equations and that it also allows risk prediction in non-White individuals. However, the major disadvantage is that it overestimates cardiovascular risk and might result in overtreatment of low-risk subjects.

## 4. Framingham Risk Score

The Framingham Risk score is one of the first predictive scores for CHD. It is based on the Framingham Heart study examinations of 1971 to 1974, which included participants from either the original Framingham study or from the initial investigation of the Framingham Offspring study [37,38]. Included subjects (n = 5345) were between 30 to 74 years old and free of CVD. All participants were followed-up for 12 years to ascertain the occurrence of CHD (angina pectoris, recognized and unrecognized MI, coronary insufficiency and CHD death). Hard CHD events included CHD death and MI. In 1998, Wilson et al. developed a sex-specific prediction algorithm to estimate 10-year CHD risk by relating the Fifth Report of the Joint National Committee on Detection, Evaluation and Treatment of High Blood Pressure blood pressure and National Cholesterol Education Program cholesterol categories with age, the presence of T2DM and smoking [39]. In 2008, D’ Agostino et al., based on a larger cohort of Framingham study, formulated a new sex-specific risk function tool that assessed not only the 10-year probability of CHD events, but also the risk for a first cardiovascular event (CHD, stroke, intermittent claudication and congestive heart failure) [40]. Although many concerns have been raised regarding the applicability and validity of this risk tool in different and diverse populations [41,42], many studies have validated it in other populations [43,44,45]. Other versions of Framingham risk score have also been developed, including the Lifetime Framingham CVD Risk Score at 50 years of age and the 30-year Framingham cardiovascular risk score [46,47].

Even though the Framingham risk score is of the first predictive scores for CHD, it has been outdated by the introduction of the Pooled Cohort Equations, which incorporates the Framingham study cohort. Therefore, the use of the Framingham risk score is not currently recommended.

## 5. Assign Risk Score

Assign risk score was formulated to estimate the 10-year risk of cardiovascular events in subjects without established CVD by adding social deprivation and family history to the risk factors including in the Framingham score (sex, age, T2DM, smoking, TC and SBP) [48,49,50]. It is based on the Scottish Heart Health Extended Cohort and is easily accessible online [48,49,50]. According to this score, patients with a score higher than 20% are considered to be at high risk [48,49,50]. The Assign score has been validated in comparison to Framingham and QRISK, and slightly outperformed the former [51,52]. Even though the Assign risk score might be useful in subjects living in Scotland, it has not been externally validated in other populations, and therefore should not be used outside Scotland.

## 6. QRISK3 Score

The QRISK3 score was developed in 2017 and updated the QRISK2 algorithm which was published in 2008 and was the standard of care risk tool for prediction of 10-year risk for cardiovascular events in England [53,54]. This sex-specific tool was derived from a cohort of 2.67 million people and includes all risk factors included in the QRISK2 model (age, ethnicity, social deprivation, SBP, body mass index, TC/HDL-C ratio, smoking, family history of CHD in a first-degree relative younger than 60 years, T2DM, treated hypertension, rheumatoid arthritis, atrial fibrillation and stage 4 or 5 chronic kidney disease) along with 8 additional risk variables which were identified as possible risk factors of CVD in other studies [53,55,56,57,58]. These variables are migraine, corticosteroid use, systemic lupus erythematosus, treatment with atypical antipsychotic medications, severe mental illness, erectile dysfunction, and variability of blood pressure [53]. This model can predict with high precision the 10-year risk of cardiovascular events in the English population aged between 25 to 84 years. Although the QRISK2 score tool was validated in non-English populations and appeared to be accurate [59,60,61], validation studies of the QRISK3 score are yet to be performed. Accordingly, the use of the QRISK3 score should be limited to the English population.

## 7. Prospective Cardiovascular Münster (PROCAM) Risk Score

The PROCAM risk score was developed to assess the 10-year risk of an acute CHD event (fatal or non-fatal) using 8 established CHD risk factors (age, SBP, low-density lipoprotein cholesterol and HDL-C, triglycerides, presence of T2DM, family history of MI and smoking status) [62]. This simple scoring system was based on a cohort of 5000 men 35–65 years old, registered in the PROCAM study [62,63]. In 2007, the PROCAM risk score was updated to be applicable not only in men, but also in women, deriving data from a larger cohort of PROCAM study, which included both genders [64]. Furthermore, Assman et al. in 2007 formulated a 10-year prediction risk score for stroke based on a smaller cohort of the PROCAM study, including five risk factors (sex, age, SBP, smoking status, and presence of T2DM) [64]. Similarly with the Assign risk score and the QRISK3 score, the PROCAM risk score has not been validated in non-German populations and therefore should not be used outside Germany, even though it might be useful in this country instead of the SCORE.

## 8. CUORE Risk Score

The CUORE risk score is the national cardiovascular risk score in Italy and predicts the 10-year risk for CHD and cerebrovascular events. This score was developed from 12 Italian cohorts of 25,000 men and women, 35–69 years-old, without established CVD. It encompasses 8 established risk factors for CVD (SBP, age, TC, HDL-C, presence of T2DM, treatment for hypertension, smoking) [65]. It was created in order to depict more accurately the 10-year CVD risk in the Italian population compared to other well-known European CVD risk scores such as the SCORE, which does not include T2DM as a risk factor. Again, the lack of external validation limits the use of the CUORE risk score in the Italian population.

## 9. Reynolds Risk Score

In 2007, Ridker et al. formulated the Reynolds Risk score by the data provided from a large US cohort study of 24,000 women free from CVD and T2DM with approximately 10-years follow up for CVD (incident MI, stroke, coronary revascularization, or cardiovascular death) [66]. This prediction model contained several established risk factors for CVD (age, sex, SBP, HbA_1c_ if diabetic, smoking, TC and HDL-C) and also considered high-sensitivity C-reactive protein (hsCRP) and parental history of MI before the age of 60 years [45]. Compared with the Adult Treatment Panel III prediction scores, the Reynolds risk score reclassified 40–50% of women of intermediate-risk in lower or higher risk categories with a good fitting of predicted and actual events [45]. In 2008, the Reynolds risk score for men was created based on a US cohort study of 10,724 men [67]. The major disadvantage of the Reynolds risk score is that it is derived from only two cohort studies and its external validity is questionable. Moreover, it is unclear whether hsCRP is a risk factor for CVD and whether the incorporation of hsCRP in a predictive model increases its accuracy [68,69].

## 10. Imaging Markers

Several studies reported that evaluation of coronary artery calcification (CAC) is a useful tool in the primary prevention of CVD.In the Multi-Ethnic Study of Atherosclerosis (n = 6814 subjects without established CVD), each increase in CAC score by 1 standard deviation was associated with an increase in the incidence of coronary heart disease by 260% [70]. More importantly, a systematic review of four observational studies (n = 13,969 subjects without established CVD) showed that measurement of the CAC score reclassified 14–4% of intermediate-risk patients to the high- or low-risk category [71]. Despite these advantages of CAC score, this predictive modality also has important shortcomings including a relatively high cost, exposure to radiation, limited availability and a low predictive value in young subjects, who are unlikely to have CAC. Accordingly, measurement of the CAC score has not been yet incorporated in a risk prediction equation. Notably, CAC appears to be less useful in risk prediction in women than in men [72].

Carotid intima-media thickness is another marker of subclinical atherosclerosis that has been used for risk prediction. However, in a meta-analysis of 14 population-based cohorts (n = 45,828), the addition of cIMT measurement to the Framingham risk score yielded minimal improvement in the predictive ability of the latter score [73]. In contrast, measurement of the ankle-brachial index, a marker of peripheral arterial disease, might be more useful in cardiovascular risk stratification. Indeed, in a meta-analysis of 16 cohort studies (n = 48,294), the ABI reclassified risk category according to the Framingham risk score in 19 and 36% of men and women, respectively [74].

## 11. Circulating Biomarkers and Genetics

A number of serological markers have been evaluated for their association with cardiovascular risk and whether they improve risk stratification when added to risk engines that incorporate traditional cardiovascular risk factors. Among these markers, N-terminal-pro-B-type natriuretic peptide (NT-proBNP) is one of the most promising. In a meta-analysis of 40 prospective studies (n = 95,617 subjects without established CVD), the addition of NT-proBNP to predictive models incorporating conventional risk factors substantially improved risk improvement [75]. Troponin also appears to improve the accuracy of conventional risk scores, both in men and in women [76].

Several polygenic risk scores have also been developed that include genes associated with atherogenesis [77]. These risk scores appear to improve the performance of conventional risk prediction equations [77,78]. However, cost and availability are important limitations for the wider use of these genetic scores.

## 12. Conclusions

**Figure 2:**
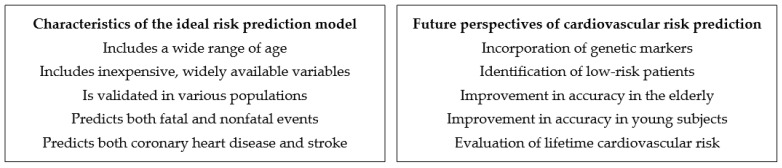
Key aspects of cardiovascular risk prediction.

Several risk scores have been developed and are being used for cardiovascular risk prediction. However, most of these risk scores have not been validated in external cohorts or were shown to over- or underestimate risk in populations other than those in which they derive. Accordingly, country-specific risk scores, where available, should be preferred for cardiovascular risk stratification. In addition, risk scores should be regularly updated with contemporary epidemiological data. Finally, it should be further evaluated whether the addition of novel cardiovascular risk markers in these scores could improve risk stratification (Figure 2).

## Figures and Tables

**Table 1:** Key characteristics of the most widely used risk prediction equations (SBP: systolic blood pressure; TC: total cholesterol; HDL-C: high-density lipoprotein cholesterol; T2DM: type 2 diabetes mellitus; MI: myocardial infarction; CHD: coronary heart disease; CVD: cardiovascular disease; LDL-C: low-density lipoprotein cholesterol; hsCRP: high-sensitivity C-reactive protein).

Risk Equation	Parameters Used to Estimate Risk	Predicted Outcome
Systematic Coronary Risk Evaluation	Age, sex, SBP, TC and smoking status	10-year risk of cardiovascular mortality
Pooled Cohort Equations Calculator	Age, sex, SBP, treatment for hypertension, TC, HDL-C, history of T2DM and smoking status	10-year risk of a nonfatal MI, CHD death and fatal or nonfatal stroke
Framingham Risk Score	Age, sex, SBP, TC, T2DM and smoking	10-year risk of a nonfatal MI and CHD death
Assign risk score	Age, sex, SBP, TC, T2DM, smoking, social deprivation and family history of CVD	10-year risk of cardiovascular events
QRISK3 score	Age, sex, SBP, TC/HDL-C ratio, T2DM, smoking status, ethnicity, social deprivation, body mass index, family history of CHD in a first-degree relative younger than 60 years, treated hypertension, rheumatoid arthritis, atrial fibrillation, stage 4 or 5 chronic kidney disease, migraine, corticosteroid use, systemic lupus erythematosus, treatment with atypical antipsychotic medications, severe mental illness, erectile dysfunction and variability of blood pressure	10-year risk of cardiovascular events
Prospective Cardiovascular Münster risk score	Age, SBP, LDL-C, HDL-C, triglycerides, presence of T2DM, family history of MI and smoking status	10-year risk of fatal or nonfatal CHD event
CUORE risk score	Age, sex, SBP, TC, HDL-C, presence of T2DM, treatment for hypertension and smoking status	10-year risk of CHD and cerebrovascular events
Reynolds Risk score	Age, sex, SBP, TC, HDL-C, HbA_1c_ if diabetic, smoking, hsCRP and parental history of MI before the age of 60 years	10-year risk of cardiovascular events

## Data Availability

Data can be found at PubMed.
